# Effect of Nanosilver Particles on *Procaspase-3* Expression in Newborn Rat Brain

**DOI:** 10.22074/cellj.2015.23

**Published:** 2015-10-07

**Authors:** Mostafa Ganjuri, Jamal Moshtaghian, Kamran Ghaedi

**Affiliations:** 1 Department of Biology, School of Sciences, University of Isfahan, Isfahan, Iran; 2 Department of Cellular Biotechnology, Cell Science Research Center, Royan Institute for Biotechnology, ACECR, Isfahan, Iran

**Keywords:** Apoptosis, Brain, Blood-Brain Barrier, *Procaspase-3*

## Abstract

**Objective:**

Nanotechnology focuses on materials having at least one dimension of less than 100 nanometers. Nanomaterials such as Nanosilver (NS) have unique physical and chemical properties such as size, shape, surface charge. NS particles are thought to in- duce neuronal degeneration and necrosis in the brain. It has been reported that NS parti- cles generate free radicals and oxidative stress which alters gene expression and induces apoptosis. This study was designed to evaluate whether the detrimental effect of NS parti- cles is through the activation of *Procaspase-3* during fetal neural development.

**Materials and Methods:**

In this experimental study, thirty Wistar female rats at day one of pregnancy were semi-randomly distributed into three groups of ten. Group 1, the control group, had no treatment. From day 1 to the end of pregnancy, groups 2 and 3 received 1 and 10 ppm NS respectively via drinking water. Newborn rats were sacrificed immediately after birth and their brains were dissected and kept frozen. Total RNA, extracted from brain homogenates, was reverse transcribed to cDNA. Quantitative real-time polymerase chain reaction (PCR) analysis was undertaken to estimate the expression level of *Procaspase-3*.

**Results:**

Developmental exposure to NS induced neurotoxicity and apoptosis. This corre- lated with a significant increase in *Procaspase-3* expression level especially at 10 ppm NS.

**Conclusion:**

The pro-apoptotic activity of NS in cells is likely to due to the dysregula- tion of *Procaspase-3*.

## Introduction

Nanoparticles (NPs) are tiny materials with onebillionth of a meter in size with specific physicochemical properties quite different from those of normal size materials of the same composition. Such properties have made NPs very attractive for industrial, commercial and medical purposes (1). Due to the very small size of NPs, their maximized surface area, exposed to any media, induces the highest possible effect per unit of weight (2). Though NPs are useful in commercial, medical and environmental sectors, their exploitative use poses harm not only to living organisms and to the environment but also to human health. Exposure to NPs can occur via air, water, food packaging materials, cosmetics and medications leading to a wide variety of toxicological effects (3). Despite a broad range of everyday use of NPs, only a very small portion of research into nanoparticles is focused on their biosafety (4). Nanosilver (NS) is superfine silver NP produced either in the form of powder or suspension in water or other liquids. NS can easily be absorbed into the cells and translocate within the human body leading to interactions with biological macromolecules (2). It has been demonstrated that NS has specific interactions with bacteria through its basic physiochemical properties. NS may combine with albumins and macroglobulins forming *Procaspase-3* silver-protein complexes which can reach the systemic circulation and then deposited in soft tissues including skin, liver, kidney, spleen, lungs and brain. Though the blood-brain barrier (BBB) plays an important role in maintaining chemical homeostasis within the brain, it has been reported that NS does penetrate this barrier (5). Studies have also revealed that NS may be transferred during pregnancy to the fetuses and accumulate in the embryonic tissues, especially the brain. Thereby NPs would be able to influence the embryonic development of the central nervous system (CNS) (6,7). On the other hand, there are numerous studies indicating that NS exposure to cell may induce DNA damage and apoptosis via oxidative stress and lipid peroxidation (8,9). This is because abnormal apoptosis has been observed in mouse embryonic fibroblasts (MEF) in response to NS exposure *in vitro* (10). The caspase family is a group of proteases involved in apoptosis. Caspases are cysteine-dependent proteases characterized by cleaving at aspartic residues. In general, caspases are localized in the cytoplasm and present as inactive proenzymes that undergo activation by proteolysis, in some cases by autocatalysis. Two apoptosomes have been identified for the activation of initiator caspases with one at the plasma membrane for activation of caspase 8 via recruitment through the death effector domain and the other in the cytoplasm for the activation of procaspase 9 via interaction with Apaf-1 and released cytochrome c of the mitocondrion. Once the initiator caspases are activated, they generate active executioner caspases (e.g. caspase 3) by cleaving their corresponding procaspases (11). To our knowledge, caspases are the main mediators of apoptosis and among capases, caspase-3 is identified to be activated both dependent and independent of mitochondrial cytochrome c release. Furthermore, adequate level of caspase-3 is essential for normal brain development (12). 

This study was thus designed to investigate whether NS exposure to pregnant female rats could induce excessive apoptotic response in brain fetuses through an increase in *Procaspase-3* expression level. We show that NS exposure is correlated with up-regulation of *Procaspase-3* which may lead to apoptosis and neuronal degeneration. 

## Materials and Methods

This study was performed as an experimental study and was approved by Graduated Office and Institutional Review Board of The University of Isfahan. 

### Use of nanosilver

The Nanocid® L-series colloidal product containing 4000 ppm NS was used (Nano Nasb Pars, Iran). This colloidal NS is water-based thus enabling it to be mixed with other water-based ingredients. Nanocid® was diluted down to the desired dose using deionized water. Transmission electron microscopy (TEM) was used to analyze the size of nanoparticles and their agglomeration state after dilution with deionized water. No changes were observed in the state of agglomeration and particle size compared with the manufacturer’s information (particle size 30 ± 4 nm). 

### Experimental animals

The permission for animal laboratory use in the experiments was obtained from the institutional review board of the University of Isfahan after considering the project and its aims. Thirty female Wistar healthy rats weighing 220 ± 20 g were obtained and kept two in a cage at the animal house of Department of Biology at University of Isfahan. In the same animal room, 15 male Wistar healthy rats weighing 250 ± 20 g were kept two in a cage. After a week of accommodation, a male rat was transferred to one of the female cages. On day 1 of pregnancy, pregnant rats were randomly distributed into three groups of 10. All animals had complete access to food and water without any limitations. Group 1 was considered as control. Groups 2 and 3 received 1 ppm and 10 ppm NS respectively via drinking water during the entire period of pregnancy. One male and one female pup per each litter with 6-8 pups on the day of birth were randomly chosen from each group. The pups were anesthetized and sacrificed. Their brains were dissected and kept frozen until further experiments. 

### RNA isolation and quantitative real time polymerase chain reaction (PCR)

Total RNA was extracted from homogenized newborn rat brains using RNX-Plus solution (CinnaGen, Iran). The extracted RNA was further purified via treatment with DNaseI (Fermentas, Germany) to remove possible contaminating genomic DNA. cDNA was synthesized from RNA samples using random hexamer and the RevertAidTM H Minus First Strand cDNA Synthesis kit (Fermentas) following the manufacturer’s protocol. Purity of the RNA extract was determined at the 260/280 nm ratio with expected values between 1.8 and 2. Quantitative real-time PCR using SYBR Green (TaKaRa, Japan) was carried out in a thermal cycler Rotor gene (Bio-RAD, USA) following the suggested protocol. The PCR mixture contained 10 µl Rotor-Gene SYBR Green PCR Master Mix (TaKaRa), 3 pM of each primer and 25 ng cDNA for each reaction in a final volume of 20 µl. Gapdh was used as an internal control. All measurements were done in triplicate. Real-time specific primer pairs for *Procaspase-3* and Gapdh were designed by the Beacon designer (Version 7.2, USA) and ordered through Metabion Company (Germany) (Table 1). Expression data were assessed and reported according to the ∆∆Ct method. 

### Statistical analysis

The results of *Procaspase-3* relative expression level in newborn rat brains are presented as mean ± standard deviation (SD). Group comparisons were conducted using ANOVA and General Linear Model. The significance level was set at P<0.05. Data were analyzed using SPSS software (Version 17). 

## Results

**Fig.1:**
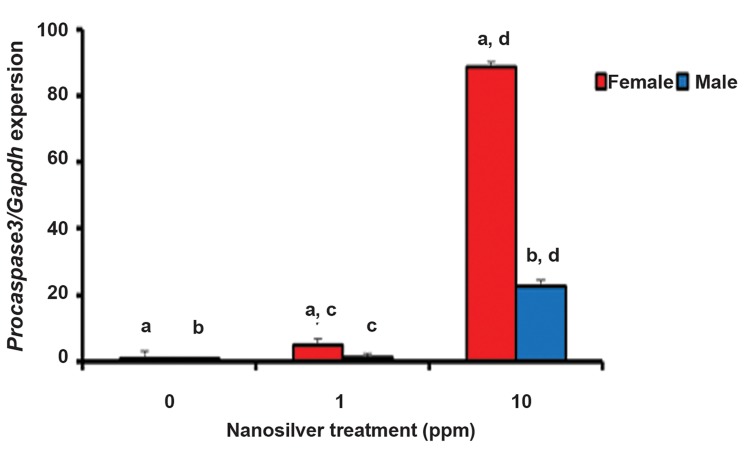
The relative expression level of *Procaspase-3* in the brains of newborn rats born to mothers exposed to 1 ppm and 10 ppm nanosilver. Similar alphabets indicate significant difference between same samples at P<0.05. For instance "a" represents significant difference between the amounts of relative expression levels of *Procaspase-3* in the control and treated groups (1 and 10 ppm) of female rats.

The results of the real-time PCR assay for evaluating the expression level of *Procaspase-3* are shown in figure 1. The expression level of *Procaspase-3* was significantly increased in the pups whose mothers had received 1 and 10 ppm of NS. This upregulation was highly significant in the 10 ppm group (approximately 88 folds in female pups and 22 folds in male pups compared with the controls). There was also a significant difference between male and female rat offsprings in the two treated groups (Table 2).

**Table 1:** Primers and conditions used for quantification of Procaspase-3 expression by real-time polymerase chain reaction (PCR)


Genes	Primer sequences (5ˊ-3ˊ)	PCR annealing temp (˚C)

*Gapdh*	F: GCATAAGATGTTTCTTCCATTTAC	58
	R: AAGAGCCTGTTCTTTAATACTTTG	
*Procaspase-3*	F: ACTTGGTTGGCTTGTTGAAG	57
	R: CTGGTATTATGGTCTGTTCCTG	


**Table 2:** Relative expression levels of *Procaspase-3* in the three groups examined


Samples	Control	1 ppm	10 ppm

Female rats	1 ± 0.65	5.3 ± 0.85	88.5 ± 0.8
Male rats	1 ± 0.17	1.4 ± 0.55	22.5 ± 0.85


The result of procaspase-3 relative expression level are presented as mean ± SD.

## Discussion

NPs can cross BBB through endothelial cells of the brain capillaries either by altering the tight junctions between those cells or by dispersing in their membranes (13). Doing both *in vitro* and in vivo studies, Rahman et al. (14) reported that NS causes a significant increase in the production of reactive oxygen species (ROS) are chemically reactive molecules containing oxygen, suggesting that NS induces neurotoxicity by generating free radicals and oxidative stress. Abnormal concentrations of ROS triggers cell commitment suicide by generating internal signals triggering death activators binding to receptors on the cell surface (15). In the embryonic stage, the brain is a highly vulnerable tissue to ROS-mediated injury due to higher oxygen consumption level, high metabolic rate associated with growth, low levels of antioxidants and protective enzymes, and high content of polyunsaturated fatty acids (16). The maintenance of cell survival is crucial for those cells which are continuously proliferating during CNS development (17). However, abnormal apoptosis can disrupt developmental processes of the brain. It has very recently been shown that prenatal exposure to NS severely affects the development of brain in neonatal rats (18). We showed a significant increase in *Procaspase-3* expression in prenatally NS treated newborn rats. This altered level of expression confirms the possible regulatory effect of NS on *Procaspase-3* expression. Procaspase activation is triggered in vertebrate cell through different pathways of apoptosis. Two well defined pathways are extrinsic and mithochondrial pathway. The initiator caspase in the extrinsic pathway is caspase-8 which proceeds the apoptosis through cleaving *Procaspase-3* and -7. Caspase-9 is the initiator caspase in the intrinsic pathway which its activation is induced upon cytochrome c release after mitochondrial outer membrane permeabilization (19). Caspase-9 acts through cleaving and activating of executioner *Procaspases-3, -6, -7*. There are numerous reports indicating induction of apoptosis under the exposure of titanium dioxide NPs especially in mouse hippocampus through increasing caspase-9 levels and activating the intrinsic apoptosis pathway (20). Similar reports have also revealed that NS-mediated apoptosis is triggered through perturbation of mitochondrial permeability (21-23). Very recently, Fatemi et al. (18) have speculated that NS may stimulate apoptosis via the intrinsic pathway in the developing brain. However, they did not measure the level of caspase- 3 in their experiment. Considering the crucial role of pro-apoptotic gene *Procaspase-3* in normal brain development, excessive *Procaspase-3* upregulation would be harmful for such processing (11). Overall, our results are in concordance with previous results (18) indicating that NS produces oxidative stress which consequently leads to apoptosis induction through an intrinsic pathway (caspase-9 activation) which is mediated by the overexpression of *Procaspase-3*. Interestingly, this dys-regulation on expression of *Procaspase-3* were significantly different in male and female newborns. It can thus be inferred that sex determinant factors may influence the severity of NS neurotoxicity. Most strikingly, we observed that increased activation of caspase-3 induces apoptosis. However, further experiments are required to clarify the mechanism of this phenomenon.

## Conclusion

We demonstrate that NS is able to induce apoptosis by dys-regulating *Procaspase-3*, however, in a sex-specific manner. Further work is therefore required to identify sex-specific interactors that result in this differential pattern.
